# Relationship of breastfeeding duration with joint pain and knee osteoarthritis in middle-aged Korean women: a cross-sectional study using the Korea National Health and Nutrition Examination Survey

**DOI:** 10.1186/s12905-020-01078-3

**Published:** 2020-09-24

**Authors:** Min-Young Kim, Hyun-Joong Kim, Je-Heon Noh, Sun-A Kim, Deok-Sang Hwang, Chang-Hoon Lee, In-Hyuk Ha

**Affiliations:** 1 Daejeon Jaseng Hospital of Korean Medicine, Daejeon, South Korea; 2 Department of Korean Medicine Gynecology, College of Korean Medicine, Kyung Hee University, Seoul, South Korea; 3 Jaseng Spine and Joint Research Institute, Jaseng Medical Foundation, 3F JS tower, 538 Gangnam-daero, Gangnam-gu, Seoul, 06110 Republic of Korea

**Keywords:** KNHANES, Lactation, Menopause, Knee osteoarthritis, Arthralgia

## Abstract

**Background:**

The effect of joint health on the quality of life of middle-aged and older women is becoming more widely recognized with the aging of the world’s population. However, the association of long-term breastfeeding with joint pain and knee osteoarthritis has not been fully examined. The aim of this study was to determine the association of prior breastfeeding duration with current joint pain and knee osteoarthritis in middle-aged Korean women.

**Methods:**

This cross-sectional study was conducted among 3454 women aged ≥50 years who underwent knee radiography and answered a questionnaire on breastfeeding and joint pain for the 5th Korea National Health and Nutrition Examination Survey (2010–2011). After adjusting for confounding sociodemographic, medical history, and obstetric and gynecologic variables, logistic regression analysis was conducted to analyze the prevalence of joint pain and knee osteoarthritis according to breastfeeding and its duration.

**Results:**

Among the 3454 participants, 298 had not breastfed and 1042, 815, and 1299 had breastfed for 1–24, 25–48, and ≥ 49 months, respectively. Of all participants, 1731 had joint pain and 739 were diagnosed with knee osteoarthritis after radiography.

Using the non-breastfeeding group as a reference, the odds ratio (OR) for joint pain among women who breastfed ≥1 month was 1.49 (95% confidence interval [CI] 1.01–2.21). As the breastfeeding duration increased, the OR of joint pain prevalence also increased (p for trend; *p* = 0.002). For knee osteoarthritis, the OR was 2.30 in the 25–48 months group (95% CI 1.09–4.86). The OR of knee osteoarthritis in the ≥49 months group was 2.17 (95% CI 1.01–4.64). Sensitivity analysis after selecting only participants aged ≥60 years showed that the prevalence of joint pain and knee osteoarthritis was more positively correlated with extended breastfeeding duration (joint pain, p for trend; *p* = 0.005) (knee osteoarthritis, p for trend; *p* = 0.012).

**Conclusions:**

Long-term feeding for more than 25 months was associated with an increased prevalence of joint pain and degenerative arthritis in Korean women aged ≥50 years.

## Background

Breast milk has species specificity and is superior to artificial compounds in providing nutrition, promoting physical development, and boosting the immune system in neonates [1]. Breastfeeding also offers numerous benefits for mothers, such as preventing depression and stress, promoting recovery to the prenatal state, and reducing the incidence of breast cancer, ovarian cancer, and type 2 diabetes mellitus [2]. Thus, WHO and UNICEF recommend exclusively breastfeeding during the first 6 months and continuing breastfeeding while providing a supplemented diet up till 2 years after birth [3]. In Korea, due to complex social obstacles such as educational opportunities for breastfeeding, social encouragement, and lack of cooperation, the exclusive breastfeeding rate, which was 52.6–40.6% in infants aged 1–4 months, significantly reduces to 9.4% in those aged 6 months. Accordingly, the Ministry of Health and Welfare is developing and promoting various policies to raise the 6-month exclusive breastfeeding rate of mothers to over 60% [4]. Worldwide, approximately 38% of newborns are exclusively breastfed for 6 months [3].

Approximately one-third of the population aged ≥65 years has osteoarthritis (OA), and this disease affects approximately 5 million individuals in Korea [5] and 100 million worldwide [6]. OA in the elderly is a recurrent factor that causes functional limitations, and is associated with a decrease in quality of life, such as disability in activities of daily living (ADL), major depression, and suicidal ideation [7, 8]. Women are more susceptible to OA. The risk of OA in the hands, knees, and hips increases after menopause [9, 10], likely owing to estrogen deficiency [11]. However, long-term estrogen deficiency can occur not only after menopause but also during breastfeeding [12]. Therefore, there may be an association between joint health in women and breastfeeding duration.

Until recently, studies of the effects of breastfeeding on musculoskeletal systems in women have focused on transient osteoporosis caused by changes in bone metabolism [13], osteoporosis linked to menopause [14] and rheumatoid arthritis [15]. The effect of joint health on the quality of life in middle-aged and older women is becoming more widely recognized with the aging of the world’s population [16]. However, the association of long-term breastfeeding with joint pain and knee OA has not been fully examined.

## Methods

### Study purpose and design

The purpose of this study was to determine whether the odds of current joint pain and knee OA change according to prior breastfeeding status and duration. We conducted post hoc analysis of cross-sectional data on Korean women.

### Study participants and examined variables

#### Participant selection

KNHANES is a sample database representing health and nutrition-related behaviors of Korean individuals nationwide. It is a statutory survey on the health behavior, chronic disease prevalence status, and food and nutrition intake status conducted by the Korea Centers for Disease Control and Prevention on a regular basis and is a government-designated statistics based on the Statistics Act of South Korea, which is actively utilized in various public health policies and academic research. As for the subjects of KNHANES, based on the latest data of Population and Housing Census of Korea each time, new demographic samples with the representability of Korean population aged 1 and over are selected, and the total participation rate of the survey is maintained at about 80%. The detailed survey guidelines and the raw data of KNHANES can be found in the website of KNHANES (http://knhanes.cdc.go.kr/) [17].

**Fig. 1:**
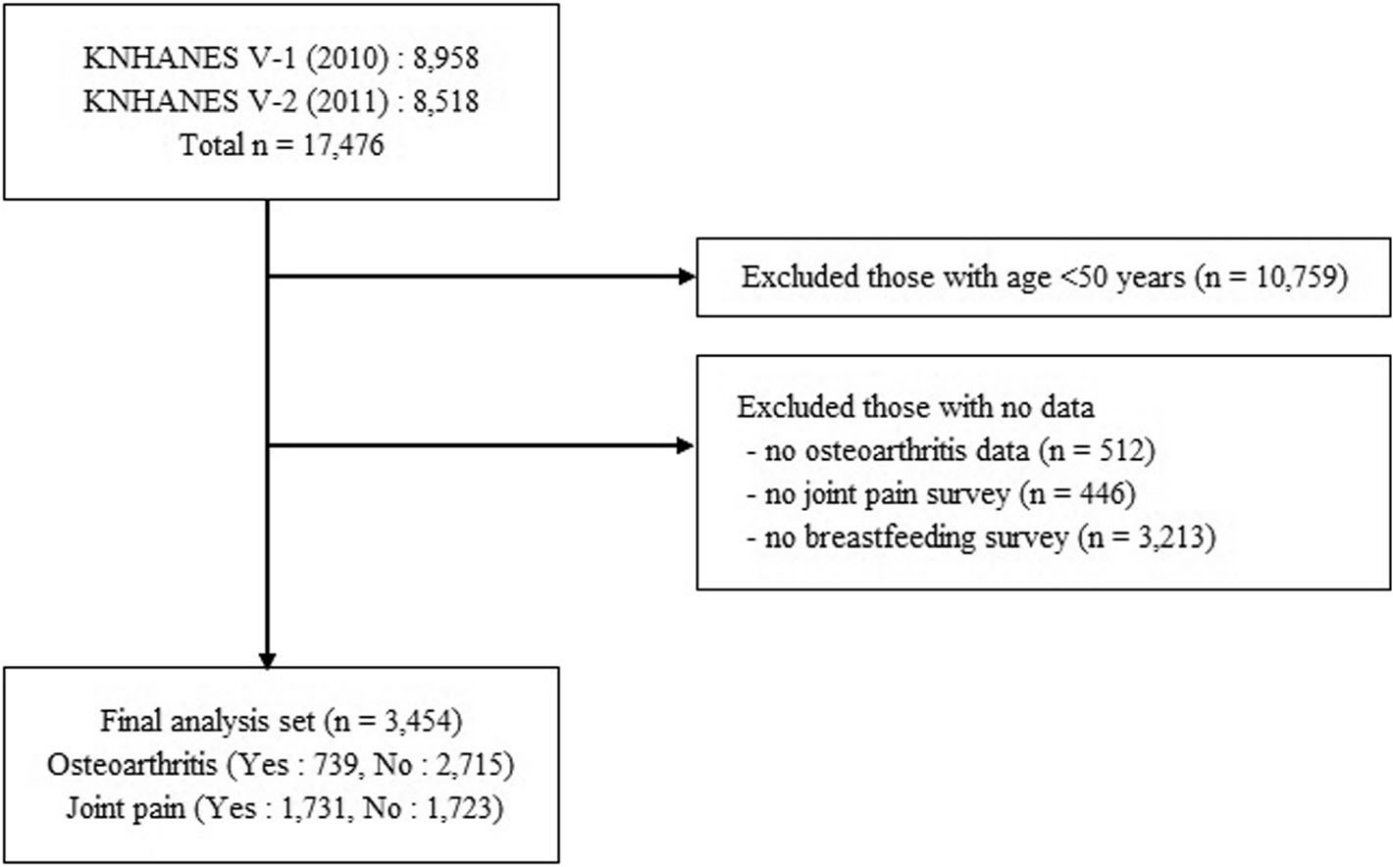
Flow chart for study population selection

The number of subjects in the 5th KNHANES was 10,938 in 2010 and 10,589 in 2011, and the actual numbers of participants were 8958 and 8518, respectively. For this study, we included women aged ≥50 years who underwent OA radiography examination and answered the self-administered questionnaire on breastfeeding. Overall, 3454 women were included (Fig. 1).

#### Breastfeeding duration

Participants who answered “no” to “(adult) Do you have breastfeeding experience of one month or longer?” in the health survey were categorized into the non-breastfeeding group. In other words, both women with no experience of breastfeeding and those with less than 1 month of breastfeeding experience were classified as non-breastfeeding. If the response was “Yes,” in the “(adult) total breastfeeding duration” item, the subject was asked to directly fill in the blank column set as two digits for the year and month, respectively, in the units of 1 month. The subject would provide the response by adding the respective breastfeeding duration in case of breastfeeding of multiple children, and in the survey, the breastfeeding duration per child was not collected. The participants were further divided into 1–24, 25–48, and ≥ 49 months breastfeeding groups. In this study, we set the breastfeeding duration that exceeds the recommended duration of 2 years as long-term breastfeeding.

#### Joint pain

The survey assessed whether respondents experienced knee, hip, and lower back pain. Those who answered “yes” to “Have you experienced knee joint pain/hip joint pain/lumbar pain for longer than 30 days in the past 3 months?” were classified into the knee joint pain, hip joint pain, and lumbar pain groups. In the overall analysis, “joint pain” was defined as the case where there was at least one joint pain in the three sites.

#### Knee osteoarthritis

Radiography for OA diagnosis was conducted for participants aged ≥50 years. As for the radiography for knee joints, in standing position, the anteroposterior view of bilateral knee joints was taken and the bilateral lateral view with the knee joint bent about 30 degrees was taken. The radiological OA diagnostic values for the knee joint were classified based on the Kellgren-Lawrence Grading Scale, as follows: 0, normal; 1, suspicion of osteoarthritis; 2, mild osteoarthritis; 3, moderate osteoarthritis; and 4, severe osteoarthritis [18]. The final grade was determined in comprehensive consideration of the findings of both joints, and as a result of the readings by three specialists in the department of OA radiology in a university hospital. When two out of three assessors had the same grade, this grade was reported, and when all three assessors reported different grades, the primary assessor’s grade was used. Those diagnosed with mild, moderate, and severe OA were considered to have knee OA in our study.

### Confounding variables

#### Sociodemographic variables

Numerical variables were used to express participant ages. Income level was divided into four categories (low, middle-low, middle-high, high) according to the average monthly equivalized household income (monthly household income/√number of household members).

#### Lifestyle variables

BMI (kg/m^2^) was used to divide participants into three groups: underweight (< 18.5), normal (18.5–24.9), and overweight (≥25) [17]. Based on smoking status, participants were classified as non-smokers (never smoked), ex-smokers, and current smokers. Second-hand smoke exposure was defined as “exposure to indoor second-hand smoke at the workplace or in the house for one hour or longer per day.” Based on alcohol consumption, participants were divided into three groups: less than once a month, 1–4 times a month, and ≥ 5 times or more per month.

The level of daily activities was classified into resting, light activities, moderate or intense activities according to the amount of physical activities performed in the week before the survey. For the moderate physical activities, the subjects who had “performed moderate physical activities requiring slightly more effort than usual and slightly accelerating heart rate for more than 30 minutes each time for more than 5 days a week” were counted, and for intense physical activities, the subjects who had “performed intense physical activities requiring considerably more effort than usual and considerably accelerating heart rate for more than 30 minutes each time for more than 5 days a week” were counted. Those who “Conduct moderate or intense physical activities as well as walking for at least 30 minutes once a day for more than 5 days a week” were categorized into the moderate or intense activities group, and those who did neither were categorized into the resting group. The rest of the participants were included in the light activities group.

#### Medical history

Participants indicated their current dyslipidemia, diabetes, hypertension, and osteoporosis status. In case of diabetes, impaired fasting glucose levels were also included. Hypertension was defined as “the presence of systolic blood pressure of ≥140 mmHg or diastolic blood pressure of ≥90 mmHg or treatment with medication for hypertension.”

#### Obstetric and gynecological variables

The numbers of natural birth, cesarean section, and preterm birth were combined to obtain the total number of children, whereas the numbers of spontaneous and induced miscarriages were combined to obtain the number of miscarriages. For the question on menstruation, among the responses of 1. Before menstruation, 2. Yes, 3. No, 8. Non-applicable (under 10 years old), 9. Unknown, those who responded with “2. Yes” were defined as non-menopausal women and those responded with “3. No” or “9. Unknown” were classified as menopausal women. Women who took hormone supplementation were checked, and the groups were categorized into 1–11 and ≥ 12 months.

#### Data analysis

KNHANES uses stratified cluster sampling and weighted values. In this study, complex sampling design analysis was conducted using stratified, cluster, and weighted variables. All data analyses were performed using SAS version 9.3 (SAS Institute Inc., Cary, NC, USA).

The participants’ sociodemographic, lifestyle, medical history, and obstetric and gynecological characteristics were analyzed according to breastfeeding duration. The complex samples general linear model and Rao-Scott chi-squared test were conducted to compare continuous and categorical variables among the non-breastfeeding (women with < 1 month of breastfeeding experience) group and the 1–24, 25–48, and ≥ 49 months breastfeeding groups. The missing values of the corrected confounding variables were excluded in analysis.

Using the non-breastfeeding group as the reference group, logistic regression models adjusting for confounders were used to calculate the odds of having the outcome (joint pain or degenerative knee OA) for subjects who breastfed ≥1 month. In addition, the breastfeeding group was further classified into 1–24 months breastfeeding group, 25–48 months breastfeeding group, and ≥ 49 months breastfeeding group. Using the non-breastfeeding group as the reference group, logistic regression models were performed to calculate the odds of having the outcome for subjects in 1–24 months breastfeeding, 25–48 months group, and ≥ 49 months breastfeeding groups.

The confounding variables include age, BMI, household income, smoking, alcohol consumption, physical activity, diabetes, hypertension, children, abortion, menopausal status, hormone replacement therapy. The first regression model adjusted only for age (Model 1), and the second regression model adjusted for all confounding variables including age (Model 2). In addition, we calculated p for trend to determine whether OR for joint pain and degenerative knee OA prevalence significantly increased with an increase in breastfeeding duration.

As knee OA is largely affected by age [19], and the differences in mean age among the breastfeeding groups were fairly large, a sensitivity analysis was conducted after re-selecting the participants aged ≥60. The OR and 95% CIs of knee OA were calculated in groups aged ≥60 years after adjusting for the same confounding variables. In all tests, *p* < 0.05 indicated statistical significance.

## Results

### Main results

Among the 3454 women aged ≥50 years, 298 were included in the non-breastfeeding group, and 1042, 815, and 1299 were included in the 1–24, 25–48, and ≥ 49 months breastfeeding groups, respectively. Among those who answered the questionnaire, 1731 participants had joint pain and 739 had knee osteoarthritis diagnosed by radiography (Table 1).

**Table 1:** Characteristics of the study population according to duration of breastfeeding above 50 years (*n* = 3454)

Variables		Duration of breastfeeding (months)				
	All women	None	1–24 mths	25–48 mths	≥49 mths	***P***-value^**a**^
	(*n* = 3454)	(*n* = 298)	(*n* = 1042)	(*n* = 815)	(*n* = 1299)	
	n (weighted %)	n (weighted %)	n (weighted %)	n (weighted %)	n (weighted %)	
**Age (years) (mean ± sd)**	3454	57.5 ± 7.6	56.5 ± 6.5	61.6 ± 7.5	70.7 ± 8.4	<.001
**Household income**	3417					
Low	1184	67 (23)	199 (19.3)	241 (29.8)	677 (52.8)	<.001
Middle-low	850	75 (25.7)	262 (25.3)	226 (28)	287 (22.4)	
Middle- High	674	58 (19.9)	251 (24.3)	190 (23.5)	175 (13.6)	
High	709	92 (31.5)	322 (31.1)	151 (18.7)	144 (11.2)	
**Smoking status**	3453					
Never smoker	2668	217 (72.8)	757 (72.7)	639 (78.4)	1055 (81.3)	<.001
Ex-smoker	102	14 (4.7)	24 (2.3)	14 (1.7)	50 (3.9)	
Current smoker	131	21 (7.1)	49 (4.7)	20 (2.5)	41 (3.2)	
Indirect smoking	552	46 (15.4)	212 (20.4)	142 (17.4)	152 (11.7)	
**Drinking**	3438					
Non alcohol consumption	1789	149 (50.2)	447 (43.2)	399 (49.1)	794 (61.4)	<.001
< 1 drinking episode per month	794	79 (26.6)	252 (24.4)	224 (27.6)	239 (18.5)	
< 5 drinking episode per month	663	52 (17.5)	262 (25.3)	154 (18.9)	195 (15.1)	
≥ 5 drinking episodes per month	192	17 (5.7)	73 (7.1)	36 (4.4)	66 (5.1)	
**BMI(kg/m**^**2**^**) (mean ± sd)**	3450	23.6 ± 2.9	24 ± 3.1	24.6 ± 3.2	24.4 ± 3.4	<.001
Underweight (< 18.5)	92	8 (2.7)	16 (1.5)	15 (1.9)	53 (4.1)	<.001
Normal (18.5 ~ 24.9)	2052	197 (66.1)	688 (66.2)	443 (54.5)	724 (55.7)	
Obese (≥25)	1306	93 (31.2)	336 (32.3)	355 (43.7)	522 (40.2)	
**Children(n) (mean ± sd)**	3451	1.7 ± 1.2	2.2 ± 0.9	3 ± 0.9	4.7 ± 1.7	<.001
None	79	75 (25.2)	2 (0.2)	1 (0.1)	1 (0.1)	<.001
1–2	1262	167 (56)	834 (80)	199 (24.5)	62 (4.8)	
3–4	1395	49 (16.4)	184 (17.7)	585 (71.9)	577 (44.5)	
≥ 5	715	7 (2.4)	22 (2.1)	29 (3.6)	657 (50.7)	
**Abortion(n)**	3445					
None	975	109 (36.8)	248 (23.9)	202 (24.8)	416 (32.1)	<.001
1–2	1664	132 (44.6)	572 (55.1)	383 (47.1)	577 (44.5)	
≥ 3	806	55 (18.6)	218 (21)	229 (28.1)	304 (23.4)	
**Hormone replacement therapy (months)**	3450					
None	2945	255 (85.9)	843 (80.9)	660 (81.1)	1187 (91.5)	<.001
< 6	149	10 (3.4)	54 (5.2)	48 (5.9)	37 (2.9)	
< 12	29	3 (1)	14 (1.3)	6 (0.7)	6 (0.5)	
≥ 13	327	29 (9.8)	131 (12.6)	100 (12.3)	67 (5.2)	
**Menopause**	3452					
No	216	37 (12.4)	133 (12.8)	37 (4.6)	9 (0.7)	<.001
Yes	3236	261 (87.6)	909 (87.2)	777 (95.5)	1289 (99.3)	
**Dyslipidemia**	3454					
No	2945	250 (83.9)	888 (85.2)	695 (85.3)	1112 (85.6)	0.592
Yes	509	48 (16.1)	154 (14.8)	120 (14.7)	187 (14.4)	
**Diabetes**	3152					
No	2047	199 (71.6)	708 (72.2)	498 (65)	642 (57)	<.001
Yes	1105	79 (28.4)	273 (27.8)	268 (35)	485 (43)	
**Hypertension**	3446					
No	1645	169 (56.9)	618 (59.5)	377 (46.4)	481 (37.1)	<.001
Yes	1801	128 (43.1)	421 (40.5)	436 (53.6)	816 (62.9)	
**Knee joint pain**	3454					
No	2365	241 (80.9)	826 (79.3)	574 (70.4)	724 (55.7)	<.001
Yes	1089	57 (19.1)	216 (20.7)	241 (29.6)	575 (44.3)	
**Hip joint pain**	3454					
No	2942	264 (88.6)	939 (90.1)	711 (87.2)	1028 (79.1)	<.001
Yes	512	34 (11.4)	103 (9.9)	104 (12.8)	271 (20.9)	
**Low back pain**	3454					
No	2247	236 (79.2)	797 (76.5)	541 (66.4)	673 (51.8)	<.001
Yes	1207	62 (20.8)	245 (23.5)	274 (33.6)	626 (48.2)	
**Osteoarthritis**	3454					
No	2715	264 (88.6)	934 (89.6)	649 (79.6)	868 (66.8)	<.001
Yes	739	34 (11.4)	108 (10.4)	166 (20.4)	431 (33.2)	

^a.^ *P*-value from complex samples general linear model or Rao-Scott chi-squared test for continuous or categorical variables

As a result of analyzing the general characteristics of each weighted group, the non-breastfeeding group(31.5%) and the ≤2 years breastfeeding group (31.1%) had the greatest percentages of respondents in the high income category. The breastfeeding groups > 25 months and > 49 months had the highest percentage of respondents in the low income category. The findings indicate that the longer the breastfeeding period, the lower the income. The non-breastfeeding group showed higher current and ex-smoking rates than the breastfeeding groups, and the rate of no alcohol consumption rate was 40% or higher in all groups. The long-term breastfeeding groups (≥25 months) had high overweight rates (BMI > 25 kg/m^2^). The findings also indicate that longer the lactation period, the higher the average number of children.

The prevalence of hormone supplementation was low in the ≥49 months breastfeeding group, and the duration was short. The percentage of those having experienced menopause was high in the ≥25 months breastfeeding groups, and 99.3% of the ≥49 months breastfeeding group had experienced menopause. The prevalence of diabetes and the percentage of joint pain complaints also increased with breastfeeding duration (Table 1).

When compared to the non-breastfeeding group, the odds of having joint pain was significantly higher for women who breastfed (OR 1.49; 95% CI 1.01–2.21). The odds of having joint pain increased with increasing lactation period (p for trend; *p* = 0.002) (Table 2). When age and all other factors were adjusted for, the ≥25 and ≥ 49 months breastfeeding groups showed a higher odds of having knee OA than the non-breastfeeding group (OR 2.30 [95% CI 1.09–4.86], OR 2.17 [95% CI 1.01–4.64]) (Table 3).

**Table 2:** Association between experience of breastfeeding, duration of breastfeeding and joint pain (*n* = 3454)

Factors	Unadjusted	Model 1	Model 2
	OR (95% CI)	OR (95% CI)	OR (95% CI)
**Breastfeeding experience (dichotomized)**			
Non-breastfeed	1	1	1
Breastfeed ≥1 mth	2.26 (1.68, 3.02)^a^	1.59 (1.18, 2.16)^b^	1.49 (1.01, 2.21)^c^
**Breastfeeding duration (categorized)**			
None	1	1	1
1–24 mths	1.20 (0.87, 1.66)	1.29 (0.93, 1.79)	1.34 (0.89, 2.00)
25–48 mths	2.13 (1.51, 3.01)^a^	1.75 (1.23, 2.49)^b^	1.67 (1.07, 2.61)^b^
≥ 49 mths	4.10 (3.08, 5.46)^a^	2.08 (1.53, 2.83)^a^	1.97 (1.29, 3.02)^b^
p for trend	<.001	<.001	0.002

Model 1 was adjusted by age

Model 2 was adjusted by age, BMI, household income, smoking, alcohol consumption, physical activity, diabetes, hypertension, children, abortion, menopausal status, hormone replacement therapy

*OR* odds ratio, *95% CI* 95% confidence interval

^a^ *p* < 0.001, ^b^ *p* < 0.01, ^c^ *p* < 0.05

**Table 3:** Association between experience of breastfeeding, duration of breastfeeding and knee osteoarthritis (OA) (*n* = 3454)

Factors	Unadjusted	Model 1	Model 2
	OR (95% CI)	OR (95% CI)	OR (95% CI)
**Breastfeeding experience (dichotomized)**			
Non-breastfeed	1	1	1
Breastfeed ≥1 mth	2.41 (1.54, 3.77)^a^	1.44 (0.92, 2.27)	1.92 (0.94, 3.92)
**Breastfeeding duration (categorized)**			
None	1	1	1
1–24 mths	0.93 (0.57, 1.53)	1.05 (0.65, 1.70)	1.60 (0.79, 3.23)
25–48 mths	2.18 (1.35, 3.52)^b^	1.67 (1.03, 2.71)^c^	2.30 (1.09, 4.86)^c^
≥ 49 mths	4.37 (2.76, 6.93)^a^	1.69 (1.03, 2.78)^c^	2.17 (1.01, 4.64)^c^
p for trend	<.001	0.004	0.062

Model 1 was adjusted by age

Model 2 was adjusted by age, BMI, household income, smoking, alcohol consumption, physical activity, diabetes, hypertension, children, abortion, menopausal status, hormone replacement therapy

*OR* odds ratio, *95% CI* 95% confidence interval

^a^ *p* < 0.001, ^b^ *p* < 0.01, ^c^ *p* < 0.05

### Sensitivity analysis results

Differences in the prevalence of joint pain between the non-breastfeeding group and breastfeeding groups were more notable in women aged ≥60 years. After adjusting for all confounding variables, the odds of joint pain were 2.61 times higher in the breastfeeding groups than in the non-breastfeeding group (95% CI 1.42–4.82), and the OR increased significantly with breastfeeding duration (p for trend; *p* = 0.005) (Table 4). The odds of having knee OA also increased in those aged ≥60 years in all breastfeeding groups (OR 2.75, 95% CI 1.17–6.50). The tendency of OR to increase with breastfeeding duration was also significant (p for trend; *p* = 0.012), and the OR of knee OA in the ≥49 months breastfeeding group was 3.18 (95% CI 1.35–7.50) (Table 5). The OR reported in the results are from Model 2 which is adjusted for all confounders.

**Table 4:** Association between experience of breastfeeding, duration of breastfeeding and joint pain, above 60 years (*n* = 2102)

Factors	Unadjusted	Model 2
	OR (95% CI)	OR (95% CI)
**Breastfeeding experience (dichotomized)**		
Non-breastfeed	1	1
Breastfeed ≥1 mth	1.91 (1.23, 2.96)^b^	2.61 (1.42, 4.82)^b^
**Breastfeeding duration (categorized)**		
None	1	1
1–24 mths	1.28 (0.77, 2.11)	2.39 (1.23, 4.64)^c^
25–48 mths	1.53 (0.94, 2.48)	2.35 (1.25, 4.41)^b^
≥ 49 mths	2.33 (1.58, 3.63)^a^	3.07 (1.63, 5.79)^b^
p for trend	<.001	0.005

Model 2 was adjusted by BMI, household income, smoking, alcohol consumption, physical activity, diabetes, hypertension, children, abortion, hormone replacement therapy. (In reselection of subjects aged over 60 years, since all the subjects were in menopause stage, the variables of age and menopausal status were removed from the confounders)

*OR* odds ratio, *95% CI* 95% confidence interval

^a^ *p* < 0.001, ^b^ *p* < 0.01, ^c^ *p* < 0.05

**Table 5:** Association between experience of breastfeeding, duration of breastfeeding and knee osteoarthritis (OA), above 60 years (*n* = 2102)

Factors	Unadjusted	Model 2
	OR (95% CI)	OR (95% CI)
**Breastfeeding experience (dichotomized)**		
Non-breastfeed	1	1
Breastfeed ≥1 mth	1.47 (0.85, 2.54)	2.75 (1.17, 6.50)^b^
**Breastfeeding duration (categorized)**		
None	1	1
1–24 mths	0.87 (0.46, 1.64)	2.14 (0.85, 5.39)
25–48 mths	1.22 (0.69, 2.14)	2.71 (1.15, 6.38)^b^
≥ 49 mths	1.76 (1.00, 3.08)^b^	3.18 (1.35, 7.50)^a^
p for trend	<.001	0.012

Model 2 was adjusted by BMI, household income, smoking, alcohol consumption, physical activity, diabetes, hypertension, children, abortion, hormone replacement therapy. (In reselection of subjects aged over 60 years, since all the subjects were in menopause stage, the variables of age and menopausal status were removed from the confounders)

*OR* odds ratio, *95% CI* 95% confidence interval

^a^ *p* < 0.01, ^b^ *p* < 0.05

## Discussion

Taken together, our data showed that prior breastfeeding experience was associated with increases in current complaints of subjective joint pain and objective diagnosis of knee OA in Korean women aged ≥50. In addition, we found that the prevalence of joint pain significantly increased with a breastfeeding duration longer than 25 months.

In this study, longer breastfeeding duration was clearly associated with higher mean age. OA is greatly influenced by age [20]. Hence, during logistic regression analysis, age was the primary factor adjusted for, and participants aged ≥60 were separated and included in a sub-analysis. The results showed greater OR between breastfeeding duration and joint pain and knee OA. When compared with the total participants aged ≥50 years, the OR of knee OA in the breastfeeding groups aged ≥60 years increased from 1.49 to 2.61 (Tables 2, 4). The OR of knee OA was 1.92 in the breastfeeding groups aged ≥50 and 2.75 in the breastfeeding groups aged ≥60, showing a marked increase in older participants (Tables 3, 5).

The effects of breastfeeding on OA have not been fully elucidated. Park’s study (2017) was the first investigation of the association between breastfeeding and OA [3]. It analyzed 6783 women aged ≥50 years using 1999–2012 National Health and Nutrition Examination Survey (NHANES) data and showed an association between breastfeeding experience of > 1 month and an increased risk of OA in older women. In that study, Park considered the “intensity of activities” that induces arthritis in detail. However, variables related to exposure to female hormones that are considered risk factors of OA in women with breastfeeding experience, such as number of pregnancies and number of children, did not show a significant association with the increased risk of OA, and breastfeeding duration could not be determined from the NHANES data.

Until recently, studies on the effects of accumulated breastfeeding duration on the musculoskeletal system in women after menopause have mainly focused on changes in bone density and rheumatoid arthritis. There is still controversy over the association of breastfeeding with bone density [21]. Some studies reported that the bone density of women who are breastfeeding or have just finished breastfeeding is higher than that of non-breastfeeding women [22, 23]. However, other studies investigating bone density among postmenopausal women showed a higher incidence of osteoporosis in those with long-term breastfeeding experience [24]. In a cohort study on 500 women aged 35–55 years, long-term breastfeeding showed significant associations with a decrease in spine bone mineral density after menopause [25].

Numerous studies have reported a negative association between breastfeeding and rheumatoid arthritis. A cohort study of 121,700 women from the Nurses’ Health Study showed that the relative risk of rheumatoid arthritis was significantly lower in those with longer breastfeeding durations [15]. In a cohort study of 18,326 participants from Sweden, while the administration of oral birth control pills was not found to lower the risk of rheumatoid arthritis. Conversely, breastfeeding, in proportion to its duration, was found to lower the risk of rheumatoid arthritis [26].

There are various risk factors for knee OA, such as old age, female sex, high level of activities, consistent exercise, past knee joint injuries, and obesity [20]. In middle-aged or older women in particular, age and female hormone deficiency can simultaneously act as risk factors of joint degeneration [10]. Estrogen deficiency is also associated with OA in humans and animals [27]. Estrogen receptors are present in several cells in the joints, including cartilage cells, subchondral bone cells, and synovial cells, and the expression of estrogen receptors increases in OA patients [27–29]. Experiments using ovariectomized animal models showed that a continued state of low estrogen concentration leads to decreased intra-articular subchondral bone mass, increased interface of the subchondral cavity, and progression of severe cartilage degradation [30]. Altogether, women experience rapid joint degeneration after menopause, around the ages of 50–75 years, and show higher prevalence, frequency, and severity of OA than men [9, 20].

Estrogen levels decrease in postmenopausal women, who often complain of muscle pain and joint pain [31]. Compared with the level in men, the estrogen level in women is 3 to 10 times higher. Changes in female hormone levels due to menstrual cycles or menopause, etc. are associated with the increase in mu-opioid receptors related to the female hormone-mediated neurotransmitters for the alleviation of intracerebral pain and perception to pain, and subsequent recall of the pain experience [32]. Therefore, this has been reported to induce a higher level of temporomandibular disorders (TMDs), fibromyalgia syndrome (FMS), and migraine in women than in men [32]. In particular, in patients with migraine, it has been found that a rapid decrease in estrogen, which occurs at the beginning of the menstruation period, further increases muscle pain and joint pain, leading to periodic occurrence of headaches [33]. Also, it has been reported that when hormone replacement therapy for postmenopausal women was discontinued, there were occurrences of muscle pain and joint pain [34].

Changes in female hormones after childbirth are partially similar to the changes noted after menopause. Estradiol, which is secreted from the placenta and increases up to 100-fold during pregnancy, instantaneously decreases during childbirth along with placenta extraction, and its concentration is maintained at a low level during breastfeeding as ovulation is delayed [12]. Hence, extended breastfeeding leads to long-term estrogen deficiency [12]. The level of female hormones increases during pregnancy, but breastfeeding can be associated with sex hormone deficiency for several years after childbirth [35]. Therefore, low levels of estrogen in breastfeeding women end with the recovery of ovarian function with delactation, but it can be inferred that they have a longer period of undergoing degenerative changes in joints, such as apoptosis of osteocytes and chondrocytes, or hypertrophy of chondrocytes, creating an intracerebral environment that is more sensitive to pain detection compared with non-breastfeeding women or women with short breastfeeding experience.

The long-term breastfeeding experience in a high percentage of Korean women aged ≥60 or older can be attributed to historical and cultural circumstances in Korea. According to a breastfeeding status survey conducted by the Korea Institute for Health and Social Affairs, the breastfeeding rate was 95% in the 1960s and rapidly decreased to 46–68.9% in the 1970s [36]. In the 1960s and 1970s, when women who are currently in their 60s and 70s were likely experiencing childbirth and breastfeeding, Korea was going through rapid economic development after the Korean war, and breast milk supplements were not yet widely marketed [37]. Thus, the period before extensive economic development and the growth of the formula market likely affected long-term breastfeeding among those aged ≥60. Furthermore, the rapid transition from an extended family to nuclear family and a declining birthrate caused by rapid industrialization and modernization are also speculated to have decreased the number of children and lifetime breastfeeding duration among this cohort [38]. Since cultural characteristics such as a sedentary lifestyle in Korea can affect the prevalence of OA in the elderly population, different outcomes might be obtained in different sample groups in the future.

This study has several strengths. To our knowledge, this study is the first to investigate the association between breastfeeding duration and the prevalence of knee OA. Joint pain in old age is a strong indicator of degenerative OA, but at the same time, there are limitations that the individual perception and expression of discomfort vary widely, and the mechanism of pain also differs by individual. OA findings through X-ray are objective and basic tests for determining skeletal status and have clinical significance, but because they do not necessarily involve pain, they cannot serve as the absolute standard for application of clinical treatment by themselves [19]. This study adopted a comprehensive approach to the clinical diagnosis of degenerative knee OA by analyzing joint pain, the subjective symptom, as a dependent variable, and subdividing the disease through knee X-ray images at the same time. The self-administered questionnaire answered by participants was systematically created by skilled experts, and the study was conducted among a large-scale group representative of Korea. In addition, various confounding variables that can affect breastfeeding and the onset of joint pain and OA were adjusted for.

The study also has some limitations. This was a cross-sectional study collecting two data variables from the same period. Thus, only the association between the two variables could be determined and the cause-and-effect relationship could not, in principle, be deduced. However, as breastfeeding is often experienced by women in their 20s and 30s and OA increases on aging, it can be speculated that breastfeeding affects the onset of OA. A sensitivity analysis was additionally conducted on the older participants aged ≥60 years, who had longer duration of breastfeeding and higher prevalence of degenerative OA, and because of the epidemiological structure that prevalence increases for those aged ≥50 years, the sensitivity analysis itself, which is limited to those aged ≥60 years, may also have limitations.

The data used in this study were responses from self-administered questionnaires, and there could be individual response errors due to the nature of the survey. Additionally, as the study participants were women aged ≥50 years, their memory of breastfeeding from decades ago might have been biased. The social atmosphere that encourages breastfeeding may cause a socially desirable response (SDR) bias in which the memory is biased or exaggerated as if they had breastfed even for women with no experience of breastfeeding or those with short-term breastfeeding, and this bias must be considered in the analysis of the results [39]. However, in this survey, health behavior items such as smoking, alcohol consumption, physical activity, and breastfeeding were surveyed in a self-administered form, not in interview form, and the content of the questions was also written in a fact-oriented manner excluding value judgment. Therefore, it may be expected that SDR bias was minimized in the survey [40].

Individual differences in the subjective perception of joint pain may have also existed. Using the response in the survey, it was defined that “If the subject has felt the pain for 30 days or longer in the last 3 months, the person has joint pain.” This is a characteristic of studies utilizing the KNHANES database, and there is a limitation of not being able to reflect the various levels of pain intensity and frequency of the total population.

Despite the findings of this study, breastfeeding has species-specific superiority as a source of nutrients and immune factors that existing breastmilk substitutes cannot surpass, and it has the effect of formation of attachment between mother and infants and preventing long-term and short-term disease. Therefore, breastfeeding should not be excluded unconditionally as a result of this study, and additional research should be undertaken carefully in other cultures and races.

## Conclusion

This cross-sectional study examined the relationship between prior breastfeeding experience, current joint pain, and knee OA. After adjusting for all confounders, breastfeeding over 25 months showed a positive correlation with odds of joint pain and knee OA. The sample group of this study experienced a rapid change in breastfeeding rate along with changes in the socio-economic environment in Korea. Therefore, it is regarded that a large-scale epidemiological investigation of degenerative OA will be needed for future Korean women who are expected to show a stable breastfeeding rate.

## Supplementary information

**Additional file 1.**

## Data Availability

Original data are publicly available for free in the KNHANES website (http://knhanes.cdc.go.kr) for purposes such as academic research. The data used in this article are open access, available online at https://knhanes.cdc.go.kr/knhanes/eng/index.do.

## Abbreviations

BMI: Body mass index
CI: Confidence interval
KNHANES: Korea National Health and Nutrition Examination Survey
OR: Odds ratio
