# Supplementary material for: Relationship of breastfeeding duration with joint pain and knee osteoarthritis in middle-aged Korean women: a cross-sectional study using the Korea National Health and Nutrition Examination Survey
Source: BMC Womens Health. 2020 Sep 24;20:213. doi: 10.1186/s12905-020-01078-3 (PMC7517693; doi:10.1186/s12905-020-01078-3)
Supplement: Supplementary file 1 — Additional file 1. [file 12905_2020_1078_MOESM1_ESM.docx]

Appendix 1. Detailed analysis result for table 2

- Logistic regression analysis result for each confusion variable for joint pain (n=3,454)

|  | | | **Unadjusted** | **Model 1** | | **Model 2** |
| --- | --- | --- | --- | --- | --- | --- |
| **Factors** | | | **OR (95% CI)** | **OR (95% CI)** | | **OR (95% CI)** |
| **Breastfeeding history** | | |  |  | |  |
|  | Non-Breastfeed | | 1 | 1 | | 1 |
|  | Breastfeed ≥1 mth | | 2.26 (1.68, 3.02)^b^ | 1.59 (1.18, 2.16)^b^ | | 1.49 (1.01, 2.21)^c^ |
| Age | |  |  | 1.07 (1.06, 1.08)^a^ | | 1.05 (1.03, 1.06)^a^ |
| BMI | |  |  |  | | 1.04 (1.01, 1.08)^b^ |
| Household income | | |  |  | |  |
|  | Low |  |  |  | | 1 |
|  | Middle-low |  |  |  | | 0.64 (0.50, 0.81)^a^ |
|  | Middle- High | |  |  | | 0.53 (0.41, 0.70)^a^ |
|  | High |  |  |  | | 0.45 (0.34, 0.59)^a^ |
| Smoking status | |  |  |  | |  |
|  | Never smoker | |  |  | | 1 |
|  | Ex-smoker |  |  |  | | 1.07 (0.62, 1.85) |
|  | Current smoker | |  |  | | 1.26 (0.78, 2.03) |
|  | Indirect smoking | |  |  | | 1.02 (0.78, 1.32) |
| Drinking | |  |  |  | |  |
|  | Non alcohol consumption | |  |  | | 1 |
|  | < 1 drinking episode per month | | |  | | 0.89 (0.71, 1.11) |
|  | < 5 drinking episode per month | | |  | | 0.86 (0.68, 1.10) |
|  | ≥ 5 drinking episodes per month |  |  |  | | 0.82 (0.55, 1.22) |
| Diabetes | |  |  |  | | 0.89 (0.74, 1.08) |
| Hypertension | |  |  |  | | 1.10 (0.91, 1.34) |
| Menopause | |  |  |  | | 0.82 (0.54, 1.23) |
| Physical activity | |  |  |  |  |  |
|  | Resting |  |  |  | | 1 |
|  | Light |  |  |  | | 0.88 (0.73, 1.06) |
|  | ≥Moderate |  |  |  | | 1.29 (0.86, 1.95) |
| Hormone replacement therapy (mth) | | |  |  | |  |
|  | None |  |  |  | | 1 |
|  | <6 |  |  |  | | 1.17 (0.79, 1.74) |
|  | <12 |  |  |  | | 0.94 (0.30, 2.98) |
|  | ≥13 |  |  |  | | 1.02 (0.75, 1.39) |
| Abortion (n) | |  |  |  | |  |
|  | None |  |  |  | | 1 |
|  | 1-2 |  |  |  | | 1.29 (1.04, 1.60)^c^ |
|  | ≥3 |  |  |  | | 1.29 (0.98, 1.70) |
| Children (n) | |  |  |  | |  |
|  | None |  |  |  | | 1 |
|  | 1-2 |  |  |  | | 0.87 (0.41, 1.83) |
|  | 3-4 |  |  |  | | 1.19 (0.56, 2.50) |
|  | ≥5 |  |  |  | | 1.54 (0.71, 3.33) |

|  | | | **Unadjusted** | | **Model 1** | **Model 2** |
| --- | --- | --- | --- | --- | --- | --- |
| **Factors** | | | **OR (95% CI)** | | **OR (95% CI)** | **OR (95% CI)** |
| **Breastfeeding duration** | | |  | |  |  |
|  | None | | 1 | | 1 | 1 |
|  | 1-24 mths | | 1.20 (0.87, 1.66) | | 1.29 (0.93, 1.79) | 1.34 (0.89, 2.00) |
|  | 25-48 mths | | 2.13 (1.51, 3.01)^a^ | | 1.75 (1.23, 2.49)^b^ | 1.67 (1.07, 2.61)^c^ |
|  | ≥49 mths | | 4.10 (3.08, 5.46)^a^ | | 2.08 (1.53, 2.83)^a^ | 1.97 (1.29, 3.02)^b^ |
|  | p for trend | | <.001 | | <.001 | 0.002 |
| Age | |  |  | | 1.06 (1.05, 1.07)^a^ | 1.04 (1.03, 1.06)^a^ |
| BMI | |  |  | |  | 1.04 (1.01, 1.07)^b^ |
| Household income | | |  | |  |  |
|  | Low |  |  | |  | 1 |
|  | Middle-low |  |  | |  | 0.64 (0.50, 0.81)^a^ |
|  | Middle- High | |  | |  | 0.54 (0.42, 0.69)^a^ |
|  | High |  |  | |  | 0.46 (0.35, 0.60)^a^ |
| Smoking status | |  |  | |  |  |
|  | Never smoker | |  | |  | 1 |
|  | Ex-smoker |  |  | |  | 1.10 (0.64, 1.88) |
|  | Current smoker | |  | |  | 1.31 (0.82, 2.09) |
|  | Indirect smoking | |  | |  | 1.03 (0.79, 1.33) |
| Drinking | |  |  | |  |  |
|  | Non alcohol consumption | |  | |  | 1 |
|  | <1 drinking episode per month | | | |  | 0.87 (0.69, 1.10) |
|  | <5 drinking episode per month | | | |  | 0.86 (0.67, 1.10) |
|  | ≥5 drinking episodes per month |  |  | |  | 0.82 (0.55, 1.22) |
| Diabetes | |  |  | |  | 0.89 (0.73, 1.07) |
| Hypertension | |  |  | |  | 1.11 (0.91, 1.35) |
| Menopause | |  |  | |  | 0.81 (0.54, 1.22) |
| Physical activity | |  |  | |  |  |
|  | Resting |  |  | |  | 1 |
|  | Light |  |  | |  | 0.87 (0.72, 1.05) |
|  | ≥Moderate |  |  | |  | 1.30 (0.86, 1.97) |
| Hormone replacement therapy (mths) | | | |  |  |  |
|  | None |  |  | |  | 1 |
|  | <6 |  |  | |  | 1.16 (0.78, 1.72) |
|  | <12 |  |  | |  | 0.93 (0.29, 2.97) |
|  | ≥13 |  |  | |  | 1.03 (0.75, 1.40) |
| Abortion (n) | |  |  | |  |  |
|  | None |  |  | |  | 1 |
|  | 1-2 |  |  | |  | 1.29 (1.04, 1.59)^b^ |
|  | ≥3 |  |  | |  | 1.30 (0.98, 1.71) |
| Children (n) | |  |  | |  |  |
|  | None |  |  | |  | 1 |
|  | 1-2 |  |  | |  | 0.90 (0.43, 1.87) |
|  | 3-4 |  |  | |  | 1.06 (0.51, 2.22) |
|  | ≥5 |  |  | |  | 1.28 (0.60, 2.76) |

OR, odds ratio; 95% CI, 95% confidence interval.

Model 1 was adjusted by age

Model 2 was adjusted by age, BMI, household income, smoking, alcohol consumption, physical activity, diabetes, hypertension, children, abortion, menopausal status, hormone replacement therapy

^a^ p<0.001, ^b^ p<0.01, ^c^ p<0.05

Appendix 2. Detailed analysis result for table 3

- Logistic regression analysis result for each confusion variable for knee osteoarthritis (OA) (n=3,454)

|  | | | **Unadjusted** | **Model 1** | **Model 2** |
| --- | --- | --- | --- | --- | --- |
| **Factors** | | | **OR (95% CI)** | **OR (95% CI)** | **OR (95% CI)** |
| **Breastfeeding history** | | |  |  |  |
|  | Non-Breastfeed | | 1 | 1 | 1 |
|  | Breastfeed ≥1 mth | | 2.41 (1.54, 3.77)^a^ | 1.44 (0.92, 2.27) | 1.92 (0.94, 3.92) |
| Age | |  |  | 1.09 (1.08, 1.10)^a^ | 1.08 (1.06, 1.10)^a^ |
| BMI | |  |  |  | 1.16 (1.11, 1.20)^a^ |
| Household income | | |  |  |  |
|  | Low |  |  |  | 1 |
|  | Middle-low |  |  |  | 0.66 (0.47, 0.91)^c^ |
|  | Middle- High | |  |  | 0.62 (0.45, 0.86)^b^ |
|  | High |  |  |  | 0.51 (0.33, 0.78)^b^ |
| Smoking status | |  |  |  |  |
|  | Never smoker | |  |  | 1 |
|  | Ex-smoker |  |  |  | 0.87 (0.45, 1.69) |
|  | Current smoker | |  |  | 0.96 (0.51, 1.80) |
|  | Indirect smoking | |  |  | 1.30 (0.94, 1.80) |
| Drinking | |  |  |  |  |
|  | Non alcohol consumption | |  |  |  |
|  | <1 drinking episode per month | | |  | 1 |
|  | <5 drinking episode per month | | |  | 1.06 (0.73, 1.53) |
|  | ≥5 drinking episodes per month | | |  | 0.85 (0.52, 1.39) |
| Diabetes | |  |  |  | 0.79 (0.62, 1.02) |
| Hypertension | |  |  |  | 1.31 (1.02, 1.68)^c^ |
| Menopause | |  |  |  | 1.37 (0.64, 2.92) |
| Physical activity | |  |  |  |  |
|  | Resting |  |  |  | 1 |
|  | Light |  |  |  | 0.87 (0.66, 1.15) |
|  | ≥Moderate |  |  |  | 1.53 (0.96, 2.44) |
| Hormone replacement therapy (mths) | | |  |  |  |
|  | None |  |  |  | 1 |
|  | <6 |  |  |  | 0.86 (0.50, 1.49) |
|  | <12 |  |  |  | 0.62 (0.12, 3.22) |
|  | ≥13 |  |  |  | 0.88 (0.57, 1.37) |
| Abortion (n) | |  |  |  |  |
|  | None |  |  |  | 1 |
|  | 1-2 |  |  |  | 1.15 (0.85, 1.56) |
|  | ≥3 |  |  |  | 0.85 (0.62, 1.18) |
| Children (n) | |  |  |  |  |
|  | None |  |  |  | 1 |
|  | 1-2 |  |  |  | 0.38 (0.13, 1.12) |
|  | 3-4 |  |  |  | 0.53 (0.18, 1.55) |
|  | ≥5 |  |  |  | 0.63 (0.21, 1.88) |

|  | | | **Unadjusted** | **Model 1** | **Model 2** |
| --- | --- | --- | --- | --- | --- |
| **Factors** | | | **OR (95% CI)** | **OR (95% CI)** | **OR (95% CI)** |
| **Breastfeeding duration** | | |  |  |  |
|  | None | | 1 | 1 | 1 |
|  | 1-24 mths | | 0.93 (0.57, 1.53) | 1.05 (0.65, 1.70) | 1.60 (0.79, 3.23) |
|  | 25-48 mths | | 2.18 (1.35, 3.52)^b^ | 1.67 (1.03, 2.71)^c^ | 2.30 (1.09, 4.86)^c^ |
|  | ≥49 mths | | 4.37 (2.76, 6.93)^a^ | 1.69 (1.03, 2.78)^c^ | 2.17 (1.01, 4.64)^c^ |
|  | p for trend | | <.001 | 0.004 | 0.062 |
| Age | |  |  | 1.08 (1.07, 1.10)^a^ | 1.08 (1.06, 1.10)^a^ |
| BMI | |  |  |  | 1.15 (1.11, 1.20)^a^ |
| Household income | | |  |  |  |
|  | Low |  |  |  | 1 |
|  | Middle-low |  |  |  | 0.65 (0.47, 0.91)^c^ |
|  | Middle- High | |  |  | 0.62 (0.45, 0.86)^b^ |
|  | High |  |  |  | 0.51 (0.34, 0.79)^b^ |
| Smoking status | |  |  |  |  |
|  | Never smoker | |  |  | 1 |
|  | Ex-smoker |  |  |  | 0.90 (0.46, 1.74) |
|  | Current smoker | |  |  | 0.99 (0.53, 1.87) |
|  | Indirect smoking | |  |  | 1.31 (0.95, 1.82) |
| Drinking | |  |  |  |  |
|  | Non alcohol consumption | |  |  | 1 |
|  | < 1 drinking episode per month | | |  | 0.82 (0.60, 1.11) |
|  | < 5 drinking episode per month | | |  | 1.06 (0.73, 1.54) |
|  | ≥ 5 drinking episodes per month | | |  | 0.86 (0.52, 1.41) |
| Diabetes | |  |  |  | 0.79 (0.61, 1.01) |
| Hypertension | |  |  |  | 1.31 (1.02, 1.69)^c^ |
| Menopause | |  |  |  | 1.34 (0.63, 2.85) |
| Physical activity | |  |  |  |  |
|  | Resting |  |  |  | 1 |
|  | Light |  |  |  | 0.87 (0.66, 1.14) |
|  | ≥Moderate |  |  |  | 1.55 (0.97, 2.50) |
| Hormone replacement therapy (mths) | | |  |  |  |
|  | None |  |  |  | 1 |
|  | <6 |  |  |  | 0.84 (0.49, 1.46) |
|  | <12 |  |  |  | 0.59 (0.11, 3.29) |
|  | ≥13 |  |  |  | 0.88 (0.56, 1.37) |
| Abortion (n) | |  |  |  |  |
|  | None |  |  |  | 1 |
|  | 1-2 |  |  |  | 1.15 (0.85, 1.54) |
|  | ≥3 |  |  |  | 0.85 (0.62, 1.17) |
| Children (n) | |  |  |  |  |
|  | None |  |  |  | 1 |
|  | 1-2 |  |  |  | 0.40 (0.14, 1.17) |
|  | 3-4 |  |  |  | 0.48 (0.16, 1.40) |
|  | ≥5 |  |  |  | 0.58 (0.19, 1.74) |

OR, odds ratio; 95% CI, 95% confidence interval.

Model 1 was adjusted by age

Model 2 was adjusted by age, BMI, household income, smoking, alcohol consumption, physical activity, diabetes, hypertension, children, abortion, menopausal status, hormone replacement therapy

^a^ p<0.001, ^b^ p<0.01, ^c^ p<0.05

Appendix 3. Detailed analysis result for table 4

- Logistic regression analysis result for each confusion variable for joint pain, above 60 years (n=2,102)

|  | | | **Unadjusted** | **Model 2** |
| --- | --- | --- | --- | --- |
| **Factors** | | | **OR (95% CI)** | **OR (95% CI)** |
| **Breastfeeding history** | | |  |  |
|  | Non-Breastfeed | | 1 | 1 |
|  | Breastfeed ≥1 mth | | 1.91 (1.23, 2.96)^b^ | 2.61 (1.42, 4.82)^b^ |
| BMI | |  |  | 1.03 (0.99, 1.07) |
| Household income | |  |  |  |
|  | Low |  |  | 1 |
|  | Middle-low |  |  | 0.70 (0.53, 0.93)^c^ |
|  | Middle- High |  |  | 0.53 (0.37, 0.75)^a^ |
|  | High |  |  | 0.60 (0.40, 0.89)^c^ |
| Smoking status | |  |  |  |
|  | Never smoker |  |  | 1 |
|  | Ex-smoker |  |  | 1.92 (0.96, 3.83) |
|  | Current smoker |  |  | 1.19 (0.63, 2.24) |
|  | Indirect smoking | |  | 1.01 (0.69, 1.49) |
| Drinking | |  |  |  |
|  | Non alcohol consumption | |  | 1 |
|  | <1 drinking episode per month | |  | 0.82 (0.61, 1.12) |
|  | <5 drinking episode per month | |  | 0.71 (0.51, 0.98)^c^ |
|  | ≥5 drinking episodes per month |  |  | 1.14 (0.66, 1.97) |
| Diabetes | |  |  | 0.90 (0.71, 1.13) |
| Hypertension | |  |  | 1.15 (0.90, 1.47) |
| Physical activity | |  |  |  |
|  | Resting |  |  | 1 |
|  | Light |  |  | 0.72 (0.58, 0.91)^b^ |
|  | ≥Moderate |  |  | 1.30 (0.77, 2.21) |
| Hormone replacement therapy (mths) | | |  |  |
|  | None |  |  | 1 |
|  | <6 |  |  | 1.59 (0.93, 2.71) |
|  | <12 |  |  | 1.26 (0.27, 5.82) |
|  | ≥13 |  |  | 0.88 (0.57, 1.35) |
| Abortion (n) | |  |  |  |
|  | None |  |  | 1 |
|  | 1-2 |  |  | 1.17 (0.89, 1.54) |
|  | ≥3 |  |  | 1.16 (0.83, 1.60) |
| Children (n) | |  |  |  |
|  | None |  |  | 1 |
|  | 1-2 |  |  | 0.55 (0.21, 1.46) |
|  | 3-4 |  |  | 0.72 (0.27, 1.92) |
|  | ≥5 |  |  | 1.11 (0.41, 3.04) |

|  | | | **Unadjusted** | **Model 2** |
| --- | --- | --- | --- | --- |
| **Factors** | | | **OR (95% CI)** | **OR (95% CI)** |
| **Breastfeeding duration** | | |  |  |
|  | None | | 1 | 1 |
|  | 1-24 mths | | 1.28 (0.77, 2.11) | 2.39 (1.23, 4.64)^c^ |
|  | 25-48 mths | | 1.53 (0.94, 2.48) | 2.35 (1.25, 4.41)^b^ |
|  | ≥49 mths | | 2.33 (1.50, 3.63)^a^ | 3.07 (1.63, 5.79)^b^ |
|  | p for trend | | <.001 | 0.005 |
| BMI | |  |  | 1.03 (0.99, 1.07) |
| Household income | |  |  |  |
|  | Low |  |  | 1 |
|  | Middle-low |  |  | 0.71 (0.54, 0.95)^c^ |
|  | Middle- High |  |  | 0.54 (0.38, 0.77)^a^ |
|  | High |  |  | 0.60 (0.41, 0.90)^c^ |
| Smoking status | |  |  |  |
|  | Never smoker |  |  | 1 |
|  | Ex-smoker |  |  | 1.89 (0.95, 3.76) |
|  | Current smoker |  |  | 1.20 (0.65, 2.24) |
|  | Indirect smoking | |  | 1.02 (0.69, 1.51) |
| Drinking | |  |  |  |
|  | Non alcohol consumption | |  | 1 |
|  | <1 drinking episode per month | |  | 0.82 (0.61, 1.12) |
|  | <5 drinking episode per month | |  | 0.72 (0.52, 0.99)^c^ |
|  | ≥5 drinking episodes per month |  |  | 1.12 (0.65, 1.95) |
| Diabetes | |  |  | 0.89 (0.70, 1.12) |
| Hypertension | |  |  | 1.15 (0.90, 1.47) |
| Physical activity | |  |  |  |
|  | Resting |  |  | 1 |
|  | Light |  |  | 0.72 (0.58, 0.91)^b^ |
|  | ≥Moderate |  |  | 1.32 (0.78, 2.24) |
| Hormone replacement therapy (mths) | | |  |  |
|  | None |  |  | 1 |
|  | <6 |  |  | 1.61 (0.94, 2.76) |
|  | <12 |  |  | 1.28 (0.29, 5.69) |
|  | ≥13 |  |  | 0.90 (0.58, 1.39) |
| Abortion (n) | |  |  |  |
|  | None |  |  | 1 |
|  | 1-2 |  |  | 1.17 (0.89, 1.54) |
|  | ≥3 |  |  | 1.16 (0.83, 1.62) |
| Children (n) | |  |  |  |
|  | None |  |  | 1 |
|  | 1-2 |  |  | 0.58 (0.22, 1.56) |
|  | 3-4 |  |  | 0.70 (0.27, 1.86) |
|  | ≥5 |  |  | 0.97 (0.35, 2.67) |

OR, odds ratio; 95% CI, 95% confidence interval.

Model 2 was adjusted by BMI, household income, smoking, alcohol consumption, physical activity, diabetes, hypertension, children, abortion, hormone replacement therapy

^a^ p<0.001, ^b^ p<0.01, ^c^ p<0.05

Appendix 4. Detailed analysis result for table 5

- Logistic regression analysis result for each confusion variable for knee osteoarthritis (OA), above 60 years (n=2,102)

|  | | | **Unadjusted** | **Model 2** |
| --- | --- | --- | --- | --- |
| **Factors** | | | **OR (95% CI)** | **OR (95% CI)** |
| **Breastfeeding history** | | |  |  |
|  | Non-Breastfeed | | 1 | 1 |
|  | Breastfeed ≥1 mth | | 1.47 (0.85, 2.54) | 2.75 (1.17, 6.50)^c^ |
| BMI | |  |  | 1.12 (1.07, 1.17)^a^ |
| Household income | |  |  |  |
|  | Low |  |  | 1 |
|  | Middle-low |  |  | 0.64 (0.46, 0.89)^b^ |
|  | Middle- High |  |  | 0.66 (0.44, 0.98)^c^ |
|  | High |  |  | 0.64 (0.40, 1.03) |
| Smoking status | |  |  |  |
|  | Never smoker |  |  | 1 |
|  | Ex-smoker |  |  | 1.12 (0.57, 2.20) |
|  | Current smoker |  |  | 0.95 (0.42, 2.13) |
|  | Indirect smoking | |  | 0.98 (0.67, 1.43) |
| Drinking | |  |  |  |
|  | Non alcohol consumption | |  | 1 |
|  | <1 drinking episode per month | |  | 0.65 (0.47, 0.91)^c^ |
|  | <5 drinking episode per month | |  | 0.85 (0.57, 1.29) |
|  | ≥5 drinking episodes per month | | | 0.83 (0.48, 1.44) |
| Diabetes | |  |  | 0.88 (0.67, 1.17) |
| Hypertension | |  |  | 1.33 (0.98, 1.80) |
| Physical activity | |  |  |  |
|  | Resting |  |  | 1 |
|  | Light |  |  | 0.83 (0.63, 1.10) |
|  | ≥Moderate |  |  | 1.31 (0.78, 2.22) |
| Hormone replacement therapy (mths) | | |  |  |
|  | None |  |  | 1 |
|  | <6 |  |  | 0.73 (0.38, 1.40) |
|  | <12 |  |  | 1.39 (0.22, 8.73) |
|  | ≥13 |  |  | 0.58 (0.32, 1.04) |
| Abortion (n) | |  |  |  |
|  | None |  |  | 1 |
|  | 1-2 |  |  | 0.96 (0.70, 1.32) |
|  | ≥3 |  |  | 0.76 (0.54, 1.06) |
| Children (n) | |  |  |  |
|  | None |  |  | 1 |
|  | 1-2 |  |  | 0.33 (0.09, 1.13) |
|  | 3-4 |  |  | 0.45 (0.14, 1.47) |
|  | ≥5 |  |  | 0.71 (0.21, 2.38) |

|  | | | **Unadjusted** | **Model 2** |
| --- | --- | --- | --- | --- |
| **Factors** | | | **OR (95% CI)** | **OR (95% CI)** |
| **Breastfeeding duration** | | |  |  |
|  | None | | 1 | 1 |
|  | 1-24 mths | | 0.87 (0.46, 1.64) | 2.14 (0.85, 5.39) |
|  | 25-48 mths | | 1.22 (0.69, 2.14) | 2.71 (1.15, 6.38)^c^ |
|  | ≥49 mths | | 1.76 (1.00, 3.08)^c^ | 3.18 (1.35, 7.50)^b^ |
|  | p for trend | | <.001 | 0.012 |
| BMI | |  |  | 1.12 (1.07, 1.17)^a^ |
| Household income | |  |  |  |
|  | Low |  |  | 1 |
|  | Middle-low |  |  | 0.65 (0.46, 0.90)^c^ |
|  | Middle- High |  |  | 0.67 (0.45, 1.00)^c^ |
|  | High |  |  | 0.65 (0.40, 1.04) |
| Smoking status | |  |  |  |
|  | Never smoker |  |  | 1 |
|  | Ex-smoker |  |  | 1.12 (0.57, 2.21) |
|  | Current smoker |  |  | 0.98 (0.44, 2.17) |
|  | Indirect smoking | |  | 0.99 (0.68, 1.45) |
| Drinking | |  |  |  |
|  | Non alcohol consumption | |  | 1 |
|  | <1 drinking episode per month | |  | 0.66 (0.47, 0.91)^c^ |
|  | <5 drinking episode per month | |  | 0.86 (0.57, 1.30) |
|  | ≥5 drinking episodes per month | | | 0.82 (0.47, 1.43) |
| Diabetes | |  |  | 0.87 (0.66, 1.16) |
| Hypertension | |  |  | 1.33 (0.98, 1.79) |
| Physical activity | |  |  |  |
|  | Resting |  |  | 1 |
|  | Light |  |  | 0.83 (0.63, 1.10) |
|  | ≥Moderate |  |  | 1.33 (0.79, 2.25) |
| Hormone replacement therapy (mths) | | |  |  |
|  | None |  |  | 1 |
|  | <6 |  |  | 0.73 (0.38, 1.40) |
|  | <12 |  |  | 1.44 (0.22, 9.37) |
|  | ≥13 |  |  | 0.59 (0.33, 1.07) |
| Abortion (n) | |  |  |  |
|  | None |  |  | 1 |
|  | 1-2 |  |  | 0.96 (0.70, 1.32) |
|  | ≥3 |  |  | 0.76 (0.54, 1.06) |
| Children (n) | |  |  |  |
|  | None |  |  | 1 |
|  | 1-2 |  |  | 0.37 (0.11, 1.29) |
|  | 3-4 |  |  | 0.43 (0.13, 1.38) |
|  | ≥5 |  |  | 0.63 (0.19, 2.10) |

OR, odds ratio; 95% CI, 95% confidence interval.

Model 2 was adjusted by BMI, household income, smoking, alcohol consumption, physical activity, diabetes, hypertension, children, abortion, hormone replacement therapy

^a^ p<0.001, ^b^ p<0.01, ^c^ p<0.05
